# Post‐COVID‐19 Liver Injury: Comprehensive Imaging With Multiparametric Ultrasound

**DOI:** 10.1002/jum.15778

**Published:** 2021-07-09

**Authors:** Maija Radzina, Davis Simanis Putrins, Arta Micena, Ieva Vanaga, Oksana Kolesova, Ardis Platkajis, Ludmila Viksna

**Affiliations:** ^1^ Institute of Diagnostic Radiology Pauls Stradins Clinical University Hospital Riga Latvia; ^2^ Radiology Research Laboratory Rīga Stradiņš University Riga Latvia; ^3^ Faculty of Medicine University of Latvia Riga Latvia; ^4^ Department of Infectology Rīga Stradiņš University Riga Latvia; ^5^ Joint Laboratory of Immunology and Immunogenetics Rīga Stradiņš University Riga Latvia; ^6^ Riga East Clinical University Hospital Riga Latvia; ^7^ Department of Radiology Rīga Stradiņš University Riga Latvia

**Keywords:** COVID‐19, liver injury, multiparametric ultrasound

## Abstract

**Objectives:**

This study aimed to define patterns of liver injury after severe acute respiratory syndrome coronavirus 2 (SARS‐CoV‐2) infection using multiparametric ultrasound (mpUS) in a variable patient population with differing severities of COVID‐19.

**Methods:**

Ninety patients were enrolled into the study: 56 had SARS‐CoV‐2 3–9 months prior to enrolment; 34 served as a clinically healthy control group. All patients underwent an mpUS evaluation of the liver (elastography, dispersion and attenuation imaging). Seventy‐six patients had abdominal magnetic resonance (MR) and noncontrast enhanced thoracic computed tomography (CT) scans performed at the same day. All patients were screened for biochemical markers of liver injury.

**Results:**

Liver elasticity, viscosity, and steatosis values were significantly altered in patients after COVID‐19, with particularly higher fibrosis scores compared to the control group (*P* < .001). Increased biochemical markers of liver injury correlated with changes in mpUS (*P* < .05), but not with findings on CT or MR findings. Seventeen of 34 hospitalized patients had a moderate or severe course of the disease course with more pronounced changes in mpUS. Increased body mass index was found to influence liver injury and correlated with more severe forms of COVID‐19 (*P* < .001).

**Conclusions:**

COVID‐19 can cause liver injury observable using mpUS. More severe forms of COVID‐19 and patient obesity are related to increased values of liver damage observed. In comparison to MRI and CT, mpUS appears to be more sensitive to involvement of liver parenchyma. Further research is warranted to establish this promising method for evaluating post‐COVID‐19 liver involvement in the aftermath of the pandemic.

**Abbreviations**
2D‐SWE: 2D‐shear wave elastography
ACE2: angiotensin‐converting enzyme 2
ALT: alanine aminotransferase
AST: aspartate aminotransferase
ATI: attenuation imaging
BMI: body mass index
CRP: C‐reactive protein
CT: computed tomography
GGT: gamma‐glutamyl transferase
HU: Hounsfield units
LDH: lactate dehydrogenase
mpUS: multiparametric ultrasound
MR: magnetic resonance
NAFLD: nonalcoholic fatty liver disease
RNA: ribonucleic acid
ROI: region of interest
SARS‐CoV‐2: severe acute respiratory syndrome coronavirus 2
SWD: shear‐wave dispersion
US: ultrasound

As of April 2021, more than 140 million confirmed cases and in excess of 3 million deaths from coronavirus disease 2019 (COVID‐19) have been reported worldwide.^1^ The disease, which is caused by the novel severe acute respiratory syndrome coronavirus 2 (SARS‐CoV‐2), has caused a global upheaval unprecedented in modern times. Although some of those who become infected with COVID‐19 will do so with their health mostly unaffected,^2^ many of those who do end up having symptoms experience only mild respiratory difficulty,^3^ which has so far been the main focus of study regarding COVID‐19. With increasing spread of the disease and lasting symptoms now defined as 'long Covid',^4^ awareness of the effect COVID‐19 has on organs other than lungs has been steadily growing,^5^ with studies emerging that examine both general and organ specific medium and long‐term health effects of COVID‐19.^6^, ^7^

SARS‐CoV‐2 is an enveloped ribonucleic acid (RNA) virus with a spike protein which interacts with the primary host receptor through attaching to the angiotensin‐converting enzyme 2 (ACE2) receptor.^8^ Cells that express more ACE2 receptors are more vulnerable to SARS‐CoV‐2.^9^ ACE2 is expressed in various organ systems, including lung tissue (specifically type II alveolar cells), the nervous, cardiovascular and gastrointestinal system, kidneys, endothelium, and the liver.^10^, ^11^, ^12^, ^13^ Because of the wide range of organ systems that express ACE2, research has been carried to investigate the potential health effects that SARS‐CoV‐2 might have on the liver,^14^, ^15^ gastrointestinal tract,^16^ cardiovascular and nervous system,^17^, ^18^ kidneys,^19^ and the respiratory system.^20^ For the presented study, we focused our attention on examining the liver.

COVID‐19‐associated liver injury can be defined as any liver damage that occurs during the course and treatment of COVID‐19, with or without pre‐existing liver disease,^21^ and it might be either reversible or irreversible with prolonged deficits. In the liver, ACE2 is highly expressed in the endothelial layer of small blood vessels and in cholangiocytes, with a less significant amount expressed in hepatocytes.^22^ Due to different liver cell types expressing ACE2 in varying quantities and the liver being highly influenced by other organ systems and medications, the spectrum of potential pathological mechanisms of liver injury is broad. It includes direct cytotoxicity from active viral replication of SARS‐CoV‐2 in the liver,^23^ immune‐mediated liver damage,^10^ vascular impairment due to coagulopathy, endothelitis or cardiac congestion, respiratory failure induced hypoxic changes, drug‐induced liver injury and exacerbation of an underlying chronic liver disease.^21^, ^24^ On the cellular level COVID‐19 associated liver injury is two‐fold. Firstly, it is caused by hepatocellular damage, mainly characterized by moderate steatosis, lobular and portal inflammation, and zones of apoptosis/necrosis, which causes elevation of plasma alanine aminotransferase (ALT) and aspartate aminotransferase (AST). Secondly, it causes cholangiocellular damage that affects the bile ducts and results in a rise of gamma‐glutamyl transferase (GGT) and bilirubin fractions among others.^25^

In parallel to COVID‐19‐induced liver injury, one has to consider additional effects of underlying chronic liver disease, eg, nonalcoholic fatty liver disease (NAFLD). Obesity and other components of the metabolic syndrome have been shown to correlate with the severity of COVID‐19.^26^ NAFLD is the hepatic manifestation of the metabolic syndrome and represents a spectrum of disease ranging from hepatocellular steatosis to steatohepatitis (inflammation), through to fibrosis and finally cirrhosis.^27^ Focused studies with suitable approaches are warranted to further investigate the mechanisms of liver damage from SARS‐CoV‐2 and the role of pre‐existing comorbidities.

The spectrum of available methods ranges from invasive approaches – i.e. liver biopsy – over biochemical indicators of liver disfunction to non‐invasive procedures, i.e., diagnostic imaging. A wide range of studies has shown that liver injury caused by COVID‐19 can be assessed by using such imaging modalities as ultrasound (US), magnetic resonance (MR), and computed tomography (CT), which reveal findings consistent with liver disease, including liver fattiness (steatosis).^28^, ^29^, ^30^, ^31^, ^32^ Because liver injury in COVID‐19 is multifaceted and includes steatosis and inflammation among others, an imaging modality that has the capacity to evaluate all of these while also being relatively simple to perform would be preferable in covering all aspects of parenchymal changes. We have therefore evaluated the diagnostic scope of multiparametric ultrasound (mpUS) of the liver in this context and suggest it as an appropriate tool for the follow‐up after infection. MpUS includes objective and quantifiable measurements of steatosis by means of attenuation imaging (ATI), as well as measurements of fibrosis and liver viscosity (for a more detailed description reference the methods section). The presented study was designed to sample liver injury in patients after COVID‐19 of varying severity and with different pre‐existing comorbidities using mpUS, and to compare it with other imaging methods such as CT and MRI. To the authors' knowledge both at the time of the conception of the study and at the time of writing this publication no other research regarding the use of mpUS in evaluating COVID‐19 sequelae in the liver had been published.

## Materials and Methods

### Patients

Between October and December 2020, a cohort of 90 randomly selected patients was enrolled in a cross‐sectional prospective study. Of the 90 participants, 56 had had COVID‐19 within a time period of 3 to 9 months prior to enrolment, and 34 were a clinically healthy control group with an anti‐SARS‐CoV‐2 IgM/IgG antibody titer below the threshold needed to indicate that they could have had COVID‐19 in the past. All participants gave their informed written consent and the study was approved by the appropriate local ethics committee for biomedical research.

The following clinical data were obtained in the form of a structured questionnaire: sex, age, months since first COVID‐19 symptoms and illness duration. Information about whether patients had been hospitalized was gathered and for those, who had, illness severity was graded into three groups: mild (no pneumonia or ARDS), moderate (pneumonia, no ARDS), severe (pneumonia and ARDS). Information regarding the usage of corticosteroids during hospitalization, as well as concurrent chronic illness and used medications was also acquired. The patients were weighed and had their height measured in order to calculate body mass index (BMI), according to which patients were subdivided into four categories based on World Health Organization criteria: normal (18.5–24.9 kg/m^2^), overweight (25.0–29.9 kg/m^2^), moderately obese (30.0–34.9 kg/m^2^), severely obese (more than 35.0 kg/m^2^). Information about history of chronic or inflammatory liver diseases (hepatitis), as well alcohol use, smoking, and physical activity was also gathered.

### Laboratory Workup

To evaluate biochemical markers of liver injury, blood samples of 76 patients were collected. The analysis consisted of a full blood count and a panel of liver enzymes, as well as hemostasis markers such as prothrombin time, international normalized ratio, D‐dimers as their levels could be altered in liver damage.^33^, ^34^ Other tests to prove hepatocyte and cholangiocyte damage through hypoxia, necrosis, and other mechanisms,^24^, ^25^, ^35^, ^36^ were included: AST, ALT, lactate dehydrogenase (LDH), GGT, ferritin and bilirubin fractions. Other markers, such as C‐reactive protein (CRP) or procalcitonin were used as indicators both of inflammation and hepatocellular injury.^37^ All patients were screened for anti‐SARS‐CoV‐2 IgM/IgG antibodies.

### Imaging

All 90 patients underwent an mpUS examination and 76 patients had a thoracic CT scan, an abdominal MR exam and blood test analysis performed on the same day. The radiological examinations were interpreted by two board‐certified radiologists with 15 or more years of experience.

Abdominal MR included a standardized series of sequences for all patients: FSE T2, FSE T2 'fat sat', T1, dynamic 3D‐GRE, DWI with b values of 50, 500, 1000, 1500, ADC mapping, and MRCP. MR chemical shift imaging with IP/OOP (in‐phase/out‐of‐phase) was also used to aid in the assessment of steatosis both qualitatively (visible signal drop in OOP when compared to IP) and semi‐quantitatively by calculating fat signal percentage using the formula [*S*_IP_ − *S*_OP_]/[2 × SIP] × 100.^38^

Noncontrast thoracic CT scans with a slice thickness of 0.625 mm and HRCT reconstructions were performed on a scan area that included the whole thoracic cavity and the upper abdomen up to the renal hila. The CT scans were primarily used to evaluate lung changes after COVID‐19 but that is not within the scope of this publication, and for the purposes of this study CT scans were used solely to measure hepatic attenuation. Several criteria exist in the assessment of hepatic steatosis by means of CT and differing sources list various cut‐off points for steatosis ranging from +40 to +58 Hounsfield units (HU).^30^, ^39^ In this study, attenuation values lower than or equal to +55 HU in a region of interest (ROI) with a diameter of 1 cm within the 7th to 8th liver segment were presumed to be indicative of hepatic steatosis. The depth of the subcutaneous fat layer in the corresponding level on CT scans was also measured.

Finally, all patients underwent an ultrasound examination of the liver that included standard B‐mode evaluation, Color Doppler US of the major liver vessels and mpUS evaluation of liver parenchyma. The ultrasound exam was performed using a Canon Aplio i800 series US machine and the i8CX1 probe. A standardized imaging protocol was applied using a sub‐costal approach for B‐mode and color Doppler US, and a supine intercostal approach for the mpUS exam.

B‐mode imaging was mainly used for qualitative assessment of the liver, looking for classical signs of steatosis and fibrosis. Firstly, this included parenchymal echogenicity, with steatosis causing the liver to appear hyperechoic when compared to the echogenicity of the spleen or adjacent kidney. Secondly, parenchymal echotexture was assessed: atypical coarseness would suggest fibrotic or cirrhotic changes. Color Doppler US was applied to determine possible vascular changes indicative of fibrosis or cirrhosis such as portal vein dilation or flow reduction, or reversal.

The mpUS examination included 2D‐shear wave elastography (2D‐SWE) for quantifying fibrotic changes (pathologically increased liver stiffness correlates to decreased elasticity), reported in kPa (automatically converted from the measured shear wave propagation speed m/s^−1^ into Young's modulus). At least 5 measurements were performed in a homogeneous area of liver parenchyma to obtain median values, and measurements were considered reliable if the interquartile range/median ratio (IQR/M%) was lower than 30%. Based on the measurements the patients were stratified using the Metavir score on a 5‐point scale ranging from F0 to F4, with a cut‐off value for F1 (minimal fibrosis) starting at 7.1 kPa.^40^ Median values were used for group stratification.

Another quantitative parameter of liver damage that was evaluated was shear‐wave dispersion (SWD), which is indicative of liver viscosity, and has shown potential to be an indicator of inflammatory processes within the liver.^41^, ^42^ Ultrasound frequency influences shear wave speed and the measurement of the level of dependency of shear waves to frequency is called dispersion. This in turn is correlated to viscosity.^43^ The dispersion values were calculated automatically together with elasticity when performing 2D‐SWE and were expressed in (m/s)/kHz. Patients were stratified into four groups based on cut‐off values provided by the US system manufacturer (see Table 1).

**Table 1:** Dispersion and Attenuation Imaging Severity Groups According to Cut‐Off Values Provided by the Ultrasound Device Manufacturer

	Normal	Mild	Moderate	Severe
Dispersion, (cm/s)/kHz	<12.00	12.00–13.99	14.00–15.99	>16.00
ATI, dB/cm/MHz	<0.63	0.63–0.71	0.72–0.80	>0.81

Finally, hepatic attenuation imaging (ATI gen) was carried out. Ultrasound waves are attenuated by acoustic scattering, reflection, and absorption during their propagation in the body, increasingly so with increasing fat content within hepatocytes.^44^ The attenuation coefficient was calculated in dB/cm/MHz with group stratification according to manufacturer data (Table 1).

### Statistics

Patient subgroups were compared using Fisher's exact test with *P* < .05 defined as the level for statistical significance. For correlations of imaging findings and biomarkers we used the Spearman's and Pearson's correlation factor. Further analyses were made with multifactorial binary logistic regression. Data were collected using Microsoft Excel tables and further processed with SPSS 27.

## Results

### Patients and Imaging

**Figure 1:**
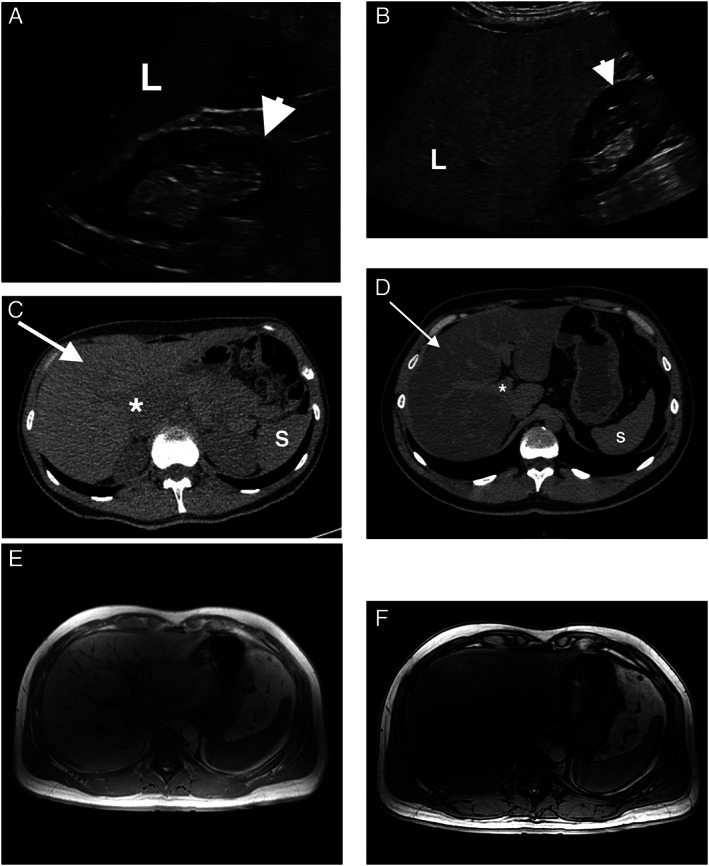
Fatty liver changes in ultrasound (US), computed tomography (CT) and magnetic resonance imaging (MRI). Normal liver parenchyma (L) is isoechoic or slightly hyperechoic in comparison with the kidney (arrowhead) or spleen in B‐mode US (**A**), whereas in case of fatty liver infiltration (steatosis) liver parenchyma becomes focally or diffusely hyperechoic as seen in (**B**). Normal liver appearance (arrow) on non‐contrast CT scans is homogenous, isodense to the spleen (S), and has a density higher than +55 Hounsfield Units (HU). Note the slightly hypodense liver vessels (*) when compared to surrounding healthy parenchyma (**C**). In case of diffuse hepatic steatosis (**D**), hepatic attenuation becomes lower than the +55 HU threshold and is hypodense when compared to the spleen. Liver vessels stand out and appear hyperdense in comparison to the parenchyma even without intravenous contrast administration. When evaluating hepatic steatosis on MRI, chemical shift imaging with IP/OOP (in‐phase/out‐of‐phase) sequences is used for qualitative assessment of liver steatosis, and in such cases, there is a visible signal drop in OOP (**F**) when compared to IP (**E**).

**Figure 2:**
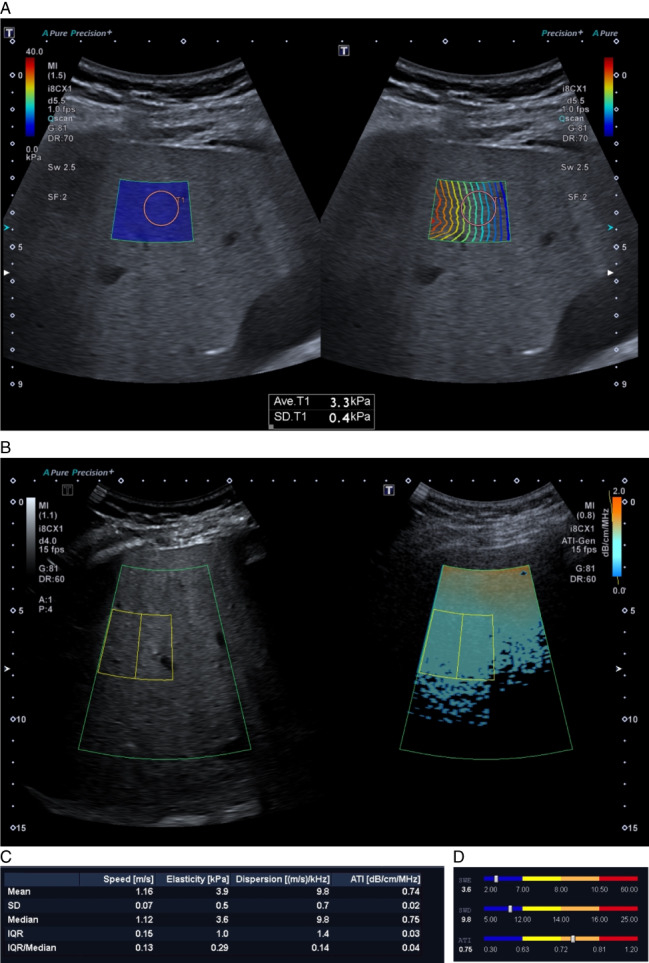
Multiparametric ultrasound (mpUS) evaluation protocol. One of the control group participants was a 55‐year‐old female with no history of liver disease. MpUS evaluation of the patient's liver included 2D‐SWE (shear‐wave elastography) with two maps in a sample box overlaid on top of the B‐mode image. These were shear wave propagation (**B**) and SWE value (**A**) maps. Proper propagation is displayed by parallel lines with constant distance between them and the ROI for making measurements (pink circle) is placed within the region where the lines are most parallel. Attenuation imaging (ATI) includes an ATI value map (**D**) and a grey scale B‐mode map (**C**), with the ROI (yellow lines) placed in the sample box below the near‐field reverberation artifact zone (orange color). *R* ^2^ values for all ATI measurements should not be lower than 0.9 (**C**, bottom left corner) and the IQR/Median proportion should always be less than 0.3 for all measurements to be reliable. The results are displayed as a bar‐chart, and in this case the patient had normal elasticity and dispersion values (SWE and SWD), however ATI values were increased: median was 0.75 dB/cm/MHz, *R* ^2^ 0.96 making the measurements reliable. This means that the patient had normal liver elasticity and viscosity, but increased steatosis values.

**Figure 3:**
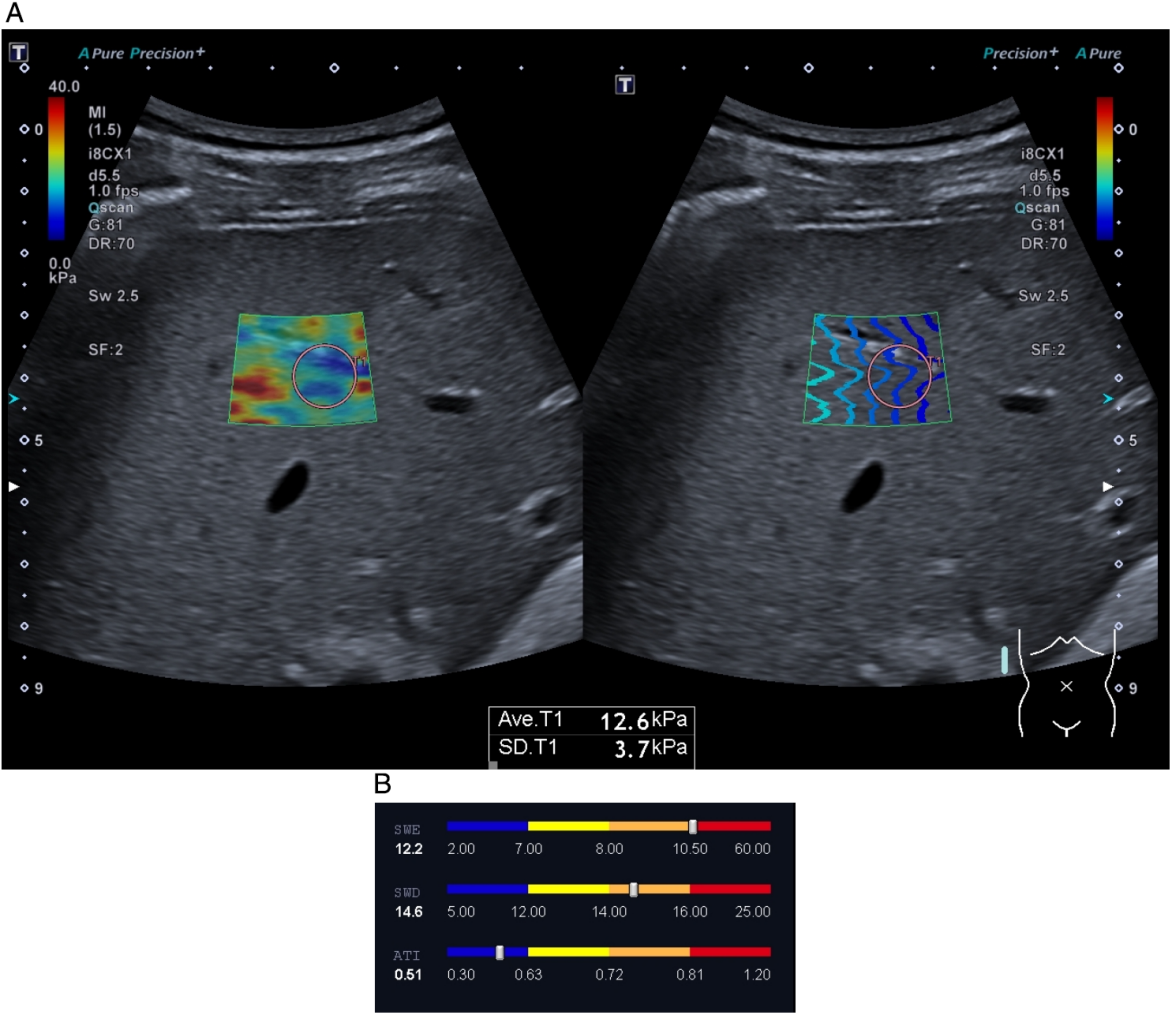
Multiparametric ultrasound evaluation protocol revealing fibrosis. (**A**) A 45‐year‐old male patient after COVID‐19 had not been hospitalized and had no residual symptoms at the time of evaluation. The patient had no previous history of liver disease, however, used excessive amounts of alcohol. SWE measurements revealed a median SWE value of 12.2 kPa‐ severely increased liver stiffness. The patient also had moderately increased SWD values, pointing toward possible liver inflammation. (**B**) ATI values were normal‐ no steatosis was observed.

**Figure 4:**
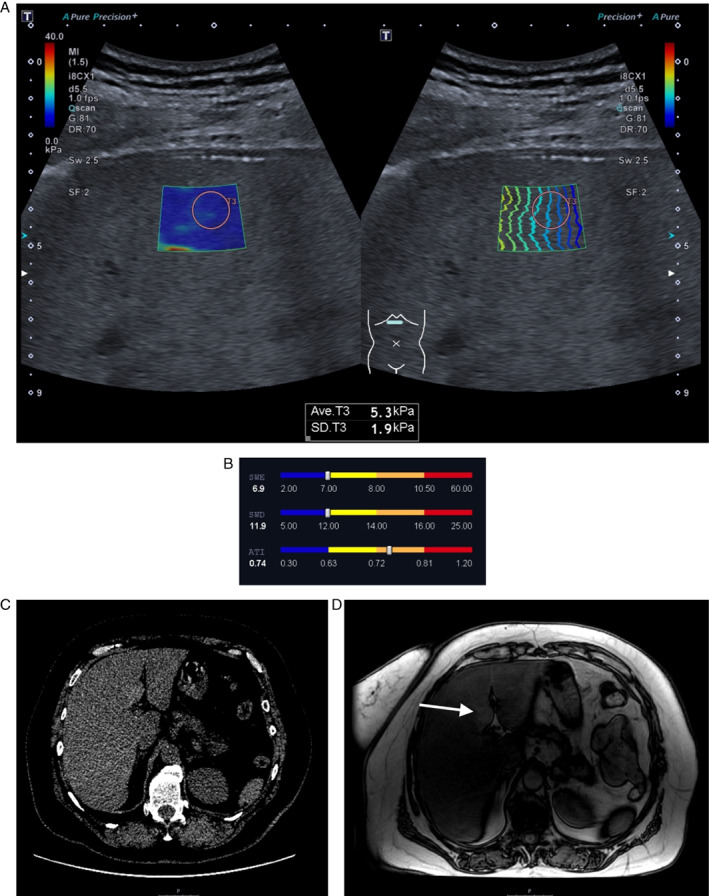
The 'full package': a case example of all imaging modalities used in the study. In a 58‐year‐old female after a severe form of COVID‐19 6 months before enrolment in the study showed consistent mpUS, CT and MRI findings indicative of hepatic steatosis in all imaging modalities. The patient had slightly increased GGT values (70 U/L), a median SWE value of 6.9 kPa, and a median SWD value of 11.9 (cm/s)/kHz, both indicating there were no detectible changes in liver stiffness or viscosity, although both values are bordering on slightly increased. ATI, however, revealed moderately increased values of 0.74 dB/cm/MHz (**A**, **B**). CT imaging revealed diffusely decreased hepatic attenuation (+35 HU), and a visible signal drop in MRI IP/OOP imaging. The patient had islets of focal fatty sparing on both non‐contrast enhanced CT (**C**) and MRI OOP (**D**) imaging (arrow), respectively exhibiting hyperdensity or hyperintensity relative to the surrounding liver parenchyma.

Of the randomly selected 90 subjects that were enrolled in the study, 56 (62%) had had COVID‐19 within a time period of 3 to 9 months prior to enrolment, and 34 (38%) were a clinically healthy control group. The different clinical information and laboratory workup data for the patient groups are shown in Table 2, whereas the main imaging findings are summarized in Table 3. To illustrate imaging examples from an mpUS examination, multiple cases are shown in Figures 1, 2, 3, 4.

**Table 2:** Research and Control Group Characteristics

	Research Group (*n* = 56)	Control Group (*n* = 34)
Age, years (range)	41.6 ± 13.4 (21–66)	39.5 ± 12.9 (19–67)
Gender; female/male, *n* (%)	28/28 (50%/50%)	21/13 (63%/38%)
Months since COVID‐19, *n*	6.4 ± 1.9 (3–9)	n.a.
Duration of COVID‐19, *n*	1.3 ± 0.6 (0.2–3)	n.a.
Hospitalization rate, *n* (%)	34 (60%)	n.a.
Disease severity, *n* (%)^a^		
Mild	13 (43%)	n.a.
Moderate	10 (33%)	n.a.
Severe	7 (23%)	n.a.
BMI, kg/m^2^ (range)	27.2 ± 4.8 (19.7–40.3)	25.0 ± 3.5 (18.4–32)
Weight group, *n* (%)		
Normal (18.5–24.9 kg/m^2^)	15 (27%)	11 (58%)
Overweight (25.0–29.9 kg/m^2^)	22 (39%)	5 (26%)
Moderately obese (30.0–34.9 kg/m^2^)	15 (27%)	3 (16%)
Severely obese (more than 35.0 kg/m^2^)	4 (7%)	0
Subcutaneous fat layer, cm (range)	1.1 ± 0.7 (0.3–3.4)	0.7 ± 0.3 (0.2–1.4)
Biochemical profile^b^		
AST, U/L [5–35 U/L] (range)	32.3 (10.0–122.0)	28.4 (15.0–32.0)
ALT, U/L [0–55 U/L] (range)	22.6 (14.0–186.0)	21.4 (16.0–84.0)
GGT, U,L [12–64 U/L] (range)	31.0 (6.0–118.0)	31.0 (13.9–101.0)
LDH, U/L [125–220 U/L] (range)	173.9 (72.0–344.0)	179.5 (130.0–236.0)
CRP, mg/L [0–5 mg/L] (range)^c^	20.5 (1.0–57.0)	3.0 (1.0–5.4)

Data presented as mean ± SD. SD, standard deviation; range (from minimum value to maximum value; n.a., not applicable (used for values irrelevant to the group in question); BMI, body mass index; AST, aspartate aminotransferase; ALT, alanine aminotransferase; GGT, gamma‐glutamyl transferase; LDH, lactate dehydrogenase; CRP, C‐reactive protein.

^a^ Data available for 30 cases.

^b^ Data available for 76 cases.

^c^ Data presented as median ± standard error.

**Table 3:** Findings in Imaging for the Research and Control Group

	Research Group (*n* = 56)	Control Group (*n* = 34)
*Imaging findings*		
Increased liver echogenicity on ultrasound, *n* (%)	5 (9%)	4 (12%)
CT liver density, HU (range)^a^	63.3 ± 6.8 (46.0–78.0)	60.4 ± 10.7 (36.0–74.0)
Study participants with steatosis indicators on MR, *n* (%)^a^	8 (15%)	3 (17%)
Mean SWE value, kPa (range)	5.06 ± 1.69 (3.0–12.2)	4.56 ± 0.90 (3.3–7.0)
Median SWE value, kPa (range)	5.05 ± 1.74 (3.0–12.2)	4.55 ± 0.78 (3.2–6.3)
Liver fibrosis group (according to Metavir)		
F0	43 (77%)	33 (97%)
F1	8 (14%)	1 (3%)
F2	3 (5%)	0
F3–4	2 (4%)	0
Mean dispersion value, (c/m)/kHz (range)	11.82 ± 1.80 (8.6–17.9)	12.01 ± 1.69 (8.6–15.6)
Median dispersion value, (c/m)/kHz (range)	11.83 ± 1.73 (8.8–18.7)	12.08 ± 1.73 (8.6–16.4)
Dispersion‐viscosity (SWD)		
Normal	37 (66%)	19 (56%)
Mildly increased	13 (23%)	12 (35%)
Moderately increased	5 (9%)	2 (6%)
Severely increased	1 (2%)	1 (3%)
Mean ATI value, dB/cm/MHz (range)	0.57 ± 0.09 (0.04–0.85)	0.57 ± 0.07 (0.45–0.79)
Median ATI value, dB/cm/MHz (range)	0.56 ± 0.09 (0.39–0.85)	0.57 ± 0.08 (0.44–0.80)
Steatosis grade		
No steatosis	47 (84%)	30 (88%)
Mildly increased	5 (9%)	2 (6%)
Moderately increased	2 (4%)	2 (6%)
Severely increased	2 (4%)	0

Data presented as mean ± SD. SD, standard deviation; range (from minimum value to maximum value; CT, computed tomography; HU, Hounsfield units; MR, magnetic resonance imaging; SWE, shear‐wave elastography; ATI, attenuation imaging.

^a^ Data available for 76 cases.

In addition to descriptive statistics, we also looked for possible relationships between different modalities, data, and disease outcomes amid many others.

Values attained in the mpUS evaluation of liver revealed statistically significantly higher SWE values comparing patients after COVID‐19 (research group patients) with the clinically healthy (control) group (5.06 ± 1.69 kPa versus 4.56 kPa ±0.90 respectively, *P* < .001). ATI values were also higher in the research group (*P* < .001), and patients in the research group were more likely to have increased SWD values when compared to their control group counterparts although the mean values between these groups did not significantly differ (*P* > .05).

**Figure 5:**
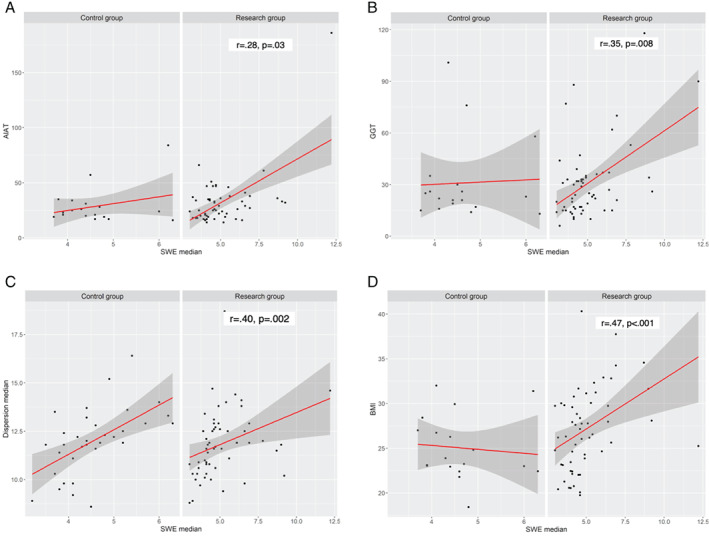
Correlations between SWE and other parameters (Spearman's correlation).

In the research group, increased liver stiffness was found to have a statistically significant positive correlation with increased levels of biomarkers of liver injury, such as ALT (*r*_s_ = 0.31, *P* = .02), GGT (*r*_s_ = 0.37, *P* = .005), as well as BMI (*r*_s_ = 0.49, *P* < .001) (see figure 5), whereas no such correlations were observed in the control group (*P* > .05). No similar relationships were observed between CT and MRI findings and the aforementioned biomarkers (*P* > .05). Steatosis (ATI) was observed to have a weak, but statistically significant (*P* < .05) correlation with biomarkers of liver injury as well.

**Figure 6:**
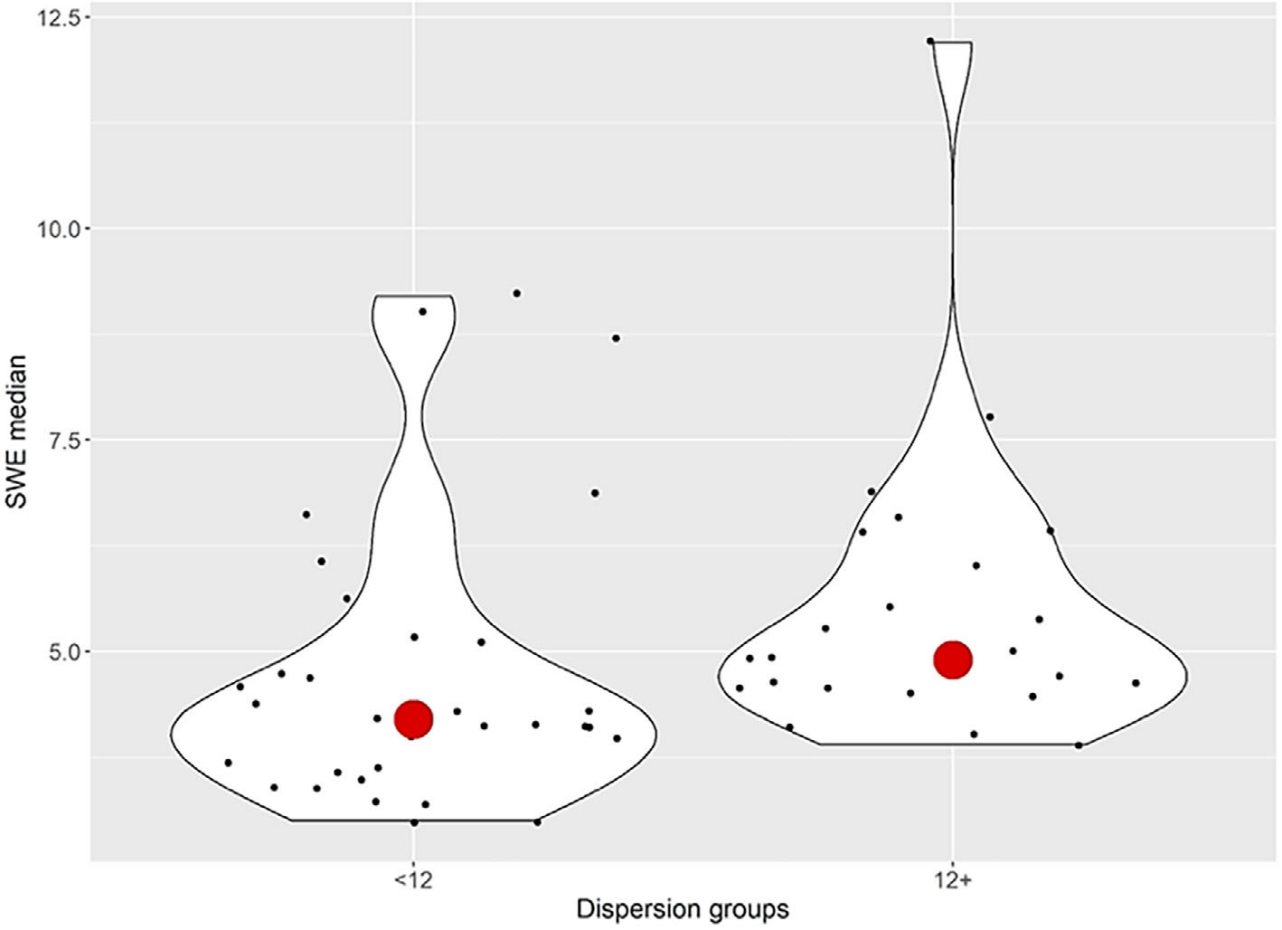
Relationship between median SWE values and dispersion groups (normal [<12] versus increased [12+] dispersion).

A mild positive correlation was found to exist between SWE and dispersion measurements, with higher SWE (median) values related to increased dispersion (OR = 1.407; *P* = .001; 95% CI 1.011–1.960). This correlation was stronger in the research group in comparison to the control group (*r*_s_ = 0.782, *P* < .001 versus *r*_s_ = 0.499, *P* < .001 respectively). (Figure 6).

**Figure 7:**
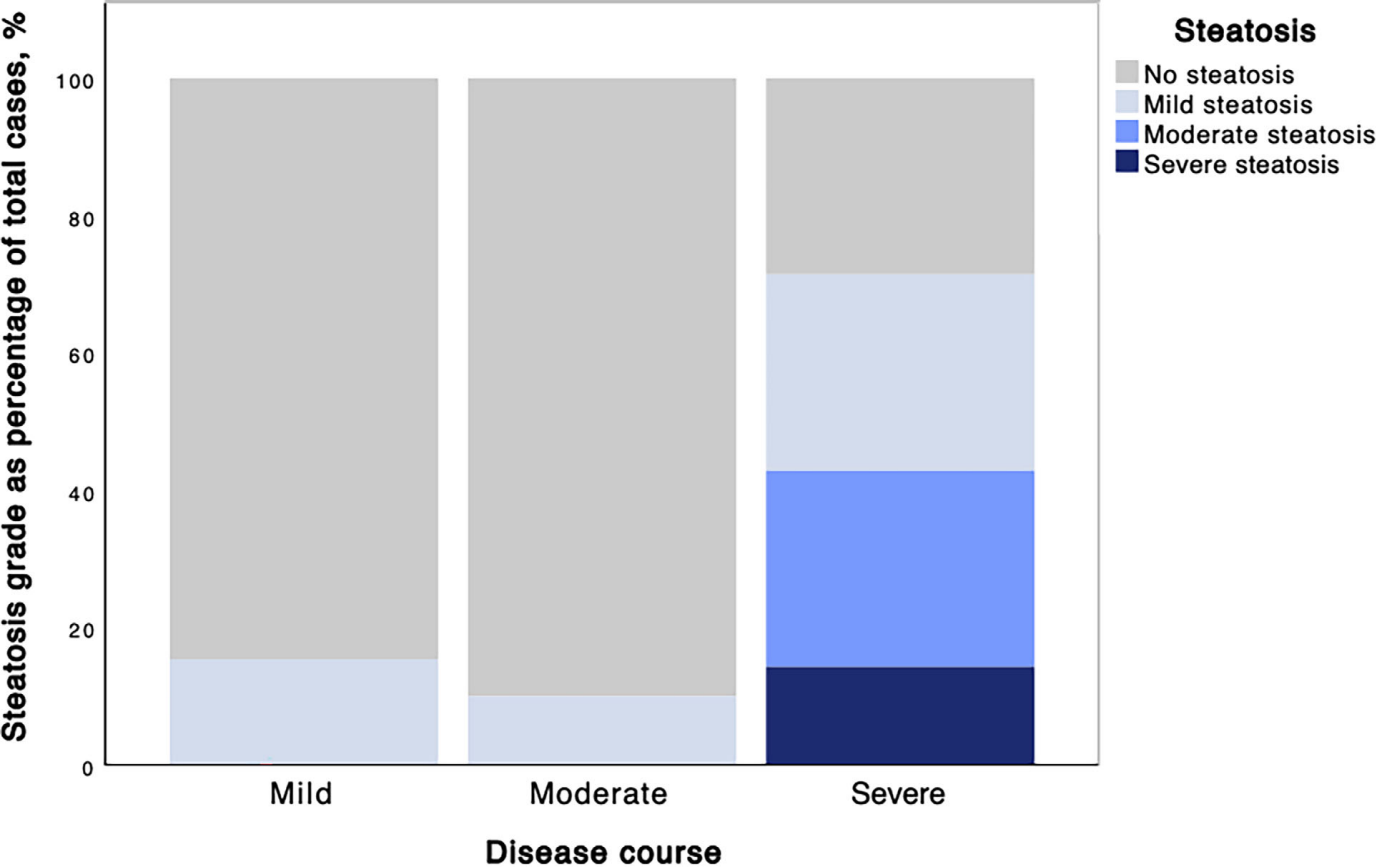
Steatosis grades groups in different disease severity.

Thirty‐four patients (60%) in the research group were hospitalized and presented with nine times higher likelihood to have steatosis (ATI) when compared to control group (*P* < .01). Research group participants who had a more severe disease course were also observed to have higher grades of steatosis (Fischer's exact test, *P* = .016) (Figure 7). Increased BMI was also correlated to increased ATI values (*P* < .001).

When comparing patients with mild and severe course of COVID‐19, study participants with increased liver echogenicity had an 8 times higher risk of severe disease course ((*P* = .001, 95% CI 2.185–29.249). The same was observed in participants with increased ATI values (five times greater risk of severe disease course, *P* = .017, 95% CI 1.536–23.438).

This also correlated to markers of inflammation, with patients who had severe disease having statistically significantly higher CRP values than patients with mild disease (*P* = .017). Research group patients had higher CRP values than their study group counterparts (*P* = .004). Obese patients (BMI ≥30) were much more likely to experience more severe COVID‐19 symptoms (*P* < .001).

Using multifactorial binary logistic regression analysis, it was found that BMI (OR 1.628, *P* = .005, 95% CI 1.158–2.289) in combination with dispersion (OR 0.302, *P* = .017, 95% CI 0.113–0.805) had a statistically significant influence on ATI scores. It was also found that CRP (OR 0.122, *P* = .003, 95% CI 0.030–0.495) when combined with median SWE values (OR 2.066, *P* = .015, 95% CI 1.151–3.706) had a statistically significant impact on increased SWD values. The results showed that higher BMI and CRP values are related to liver steatosis (BMI—OR 1.459; *P* = .001; 95% CI 1.177–1.808; CRP—OR 1.387; *P* = .026; 95% CI 1.039–1.850).

**Figure 8:**
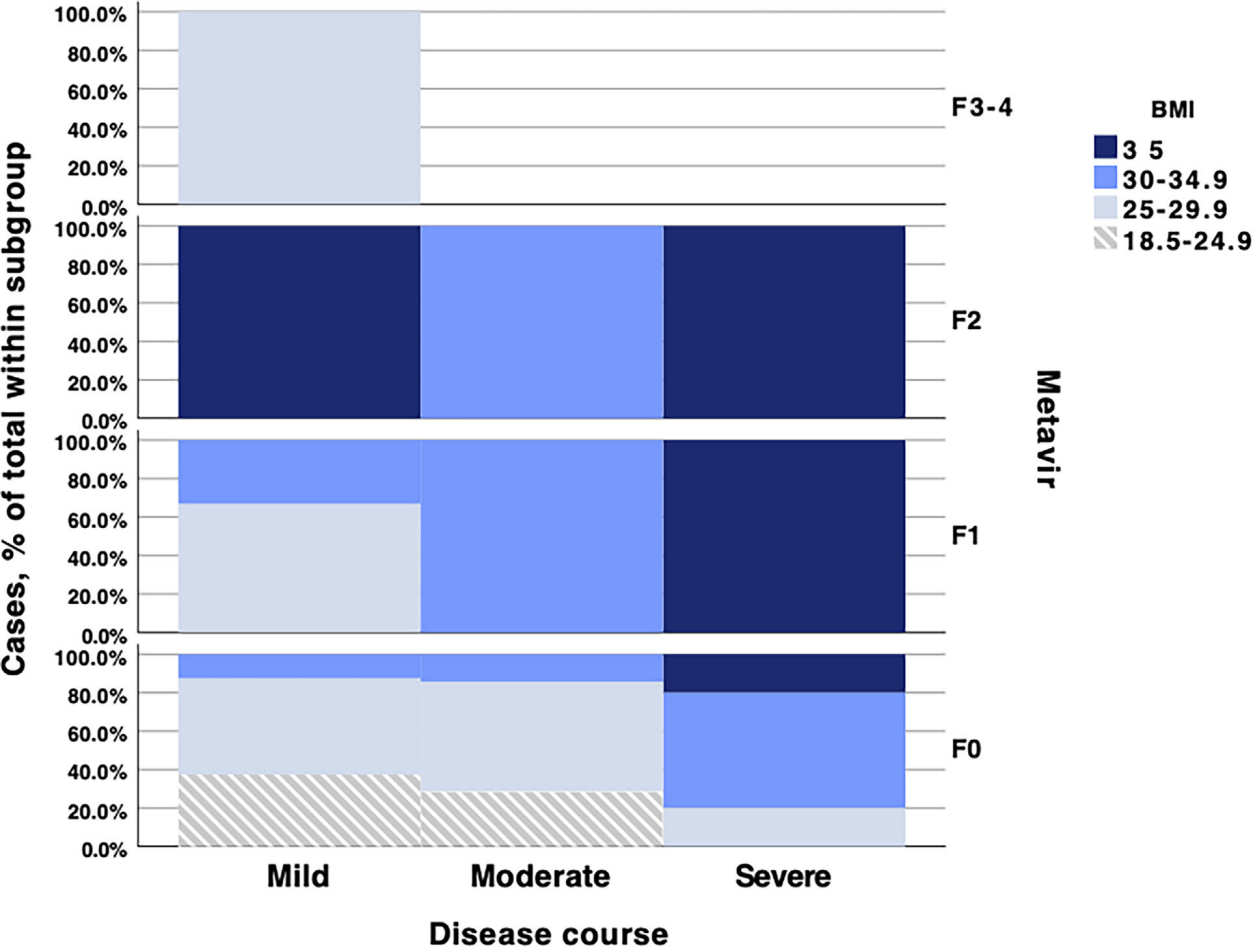
Proportion of BMI categories with differing liver stiffness scores depending on COVID‐19 severity.

One can better visualize the complex relationship between disease severity, obesity, and altered liver stiffness in Figure 8.

The increased risk of severe disease course in patients with increased ATI values correlated with MRI findings, as patients who had indicators of steatosis on MRI were more likely to experience moderate or severe disease course (Fisher's exact test, *P* = .01). US was also found to have a moderate inverse correlation to CT liver parenchymal density (*r*_s_ = −0.577; *P* = .01), with lower density on CT correlating to a more hyperechoic liver parenchyma on B‐mode ultrasound (OR 0.849; *P* = .005; 95% CI 0.758–0.951). Patients with a thicker subcutaneous fat layer had higher steatosis grades (*P* < .05), as well as higher dispersion measurements (*F* = 4.7; *P* = .035). Alcohol use, exercise, and other patient factors were not found to have meaningful impact on disease severity or imaging features.

## Discussion

Due to the main pathogenic mechanism of cell invasion of SARS‐CoV‐2, which is through binding to ACE2 receptors, the liver is among the first‐line targets of cell injury in COVID‐19 by its high expression of ACE2.^22^ The potential mechanisms of liver injury in COVID‐19 are multifaceted, including inflammation, steatosis, and biliary duct damage.^10^, ^21^, ^23^, ^24^ Liver injury caused by COVID‐19 can be assessed by using biochemical markers as well as imaging modalities such as US, MR and CT.^28^, ^29^, ^30^, ^31^, ^32^

In the presented study, multiparametric ultrasound (mpUS) evaluation of liver parenchyma in individuals after COVID‐19 revealed increased liver stiffness and steatosis (attenuation) values indicative of liver injury compared to the clinically healthy control group. The most noticeably altered parameter was increased liver stiffness represented by higher SWE values, which is consistent with findings of other studies that have evaluated specifically liver elasticity in COVID‐19.^28^ Increased liver stiffness correlated with increase in other multiparametric US indicators of liver injury‐viscosity (inflammation) and steatosis, and this correlation was stronger in patients after COVID‐19.

Increased liver stiffness also correlated well with increased levels of biomarkers of liver injury, such as ALT and GGT, which indicate underlying hepatocellular and cholangiocellular damage on a biochemical level. CRP, which has been shown to be a predictive factor of liver injury in COVID‐19 patients,^45^ was higher in the research group than the clinically healthy control group and taken together with higher SWD values regardless of BMI highlighted the underlying liver inflammation in patients after COVID‐19, while the control group showed a tendency for lower viscosity values with obesity.

Increased BMI was found to be a major factor in liver injury. BMI was found to correlate with increased liver stiffness values in the research group and was combined with more severe steatosis. The more pronounced changes in patients with increased BMI could be explained with subjects with higher BMI being more prone to severe disease course. The findings of this study also indicate that hospitalization and disease severity play a significant role in liver injury in COVID‐19 patients, and study patients with higher steatosis scores were far more likely to be hospitalized and had a more severe disease course‐ results that are concordant with findings of other studies.^46^, ^47^ The ATI measurement was the most closely related to obesity with equal importance as the indicator of steatosis, readily assessable with B‐mode US, CT and MR although the findings of the latter two did not correlate to biochemical markers of liver injury. This allows to conclude that multiparametric ultrasound might be more sensitive than CT and MRI in evaluating liver injury that is reflected both on the supracellular and cellular level prior developed cirrhosis.

The main limitation of this study is that the causality between liver injury and COVID‐19 infection is not proven, because in most cases no previous imaging data were available prior to SARS‐CoV‐2 infection. To a certain extent, it still remains unclear whether factors such as steatosis and obesity merely predispose patients to more severe COVID‐19 infections and subsequent liver damage or represent the result of more severe infection. Such further research is definitely warranted, since the applied mpUS technology appears fully suitable for this purpose and would only require a different study design to carry out a multiparametric US liver examination at a very early time of COVID‐19 diagnosis before any substantial liver damage would be expected, and a longitudinal design to follow‐up changes during hospitalization or out‐patient care.

Although most hospitalized patients undergo a thoracic CT scan that allows to measure density, as discussed earlier, liver injury in COVID‐19 shows variability beyond liver steatosis which is not fully appreciated with noncontrast enhanced CT alone. The potential diagnostic scope of dynamic contrast enhancement of the liver and spectral CT is therefore not covered with this study. MR still remains an examination that is more difficult to access and according to observed limited informativity in this study, MR would not be recommended as first‐line method for assessment of liver injury in patients after COVID‐19.

In conclusion, multiparametric ultrasound examination can reveal liver injury in patients after COVID‐19, with a wide spectrum of parenchymal changes including increased liver stiffness, increased viscosity and attenuation, indicative of fibrosis, inflammation, and steatosis, respectively. Beyond severity of the disease itself, co‐factors, such as increased BMI and pre‐existing comorbidities or medico‐toxic side‐effects may aggravate liver injury. Research on all these factors is just at its beginning, but the presented study strongly suggests that mpUS would be a highly suitable method for such future work, in particular, for the long‐term follow‐up of patients with prolonged course of the disease.
